# Management of a Hopeless Mandibular Molar: A Case Report

**Published:** 2011-02-15

**Authors:** Saeed Asgary

**Affiliations:** 1. Department of Endodontics, Iranian Center for Endodontic Research, Dental Research Center, School of Dentistry, Shahid Beheshti University of Medical Sciences, Tehran, Iran.

**Keywords:** CEM Cement, Endodontics, Tooth Reimplantation, Treatment Outcome

## Abstract

Intentional tooth reimplantation can be an alternative treatment option for teeth with poor or hopeless prognosis where coronal and surgical endodontic treatment(s) are not possible. This technique may help to restore a natural tooth to function in preference to prosthesis/implant replacements. A 38-years old male was referred to private practice with persistent chronic apical periodontitis of a previously root canal treated mandibular left first molar. A furcal perforation and distolingual cusp fracture was previously repaired and treated with amalgam (~5 years ago). In view of the patient/tooth’s limitations, intentional reimplantation was planned using CEM cement retrograde filling. Clinical and radiographic follow-up during 2 years postoperatively revealed no sign/symptoms of infection or inflammation. Moreover, periradicular healing was evident on radiographs.

## INTRODUCTION

Orthograde root canal therapy usually has a high success rate; however this treatment may at times fail [1]. In the case of failure alternative treatments, such as intentional reimplantation (IR), may be considered. This technique is an accepted treatment for cases in which orthograde and surgical endodontic (re)treatments are not feasible, or have already failed [2][3]. IR can be also considered a suitable treatment for teeth with root perforations which have difficult endodontic or surgical access [4]. Generally, IR is not recommended for teeth with periodontal disease [5][6]; however, it has recently revealed good results for treatment of periodontally involved teeth [7]. This method has also been used in the management of vertical fractures and certain anatomical malformations e.g. radicular groove [8].

A number of studies advocated that IR should be reserved as a “last resort” after exploration and/or failure of other (re)treatments options [9][10]. An alternative line of thought believes that IR is an economical and conventional technique that is of short duration and easy manipulation [11].

This method involves atraumatic tooth extraction (i.e. evading unnecessary damages to the cementum/PDL) and rapid reinsertion into the alveolus immediately after endodontic treatment/apical repair outside the oral cavity. Teeth with divergent, long and curved roots are not apt for IR since they are prone to fracture during extraction; the success of this treatment directly depends on meticulous case selection thorough clinical/radiographic evaluations [12]. Even with the aforementioned advantages, IR may be associated with inflammatory root resorption and ankylosis due to trauma to the PDL, reducing survival rate of the replanted

teeth. These complications are directly related to the time the tooth is retained extraorally for treatment; the longer the tooth is kept outside the socket, the poorer the prognosis [13].

This case report presents a mandibular first molar associated with failed RCT, large periapical lesion, and a previous furcal perforation repair treated successfully with combined IR and root-end filling/sealing using CEM cement.

## CASE REPORT

**Figure 1:**
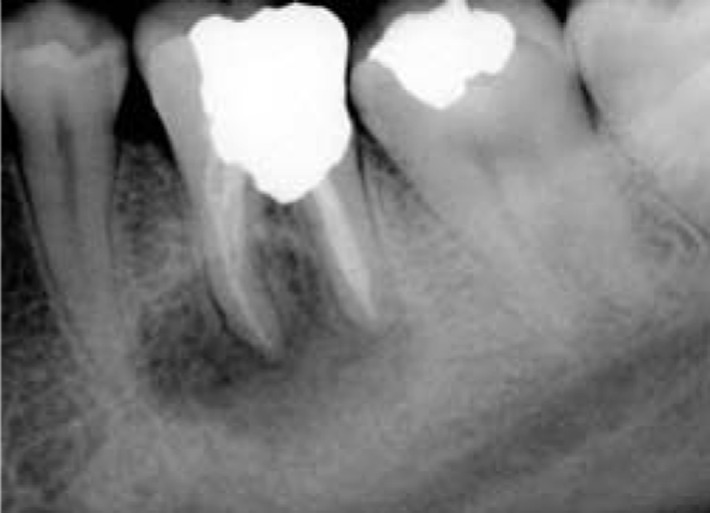
Preoperative radiograph of a mandibular first molar with a large apical lesion of the mesial root and furcal perforation repair subjected to IR.

A 38 years old male patient with a noncontributory medical history was referred to dentist with a chief complaint of periodic swelling and pain in the mandibular left molar region. The extraoral examination was unremarkable. In the intraoral examination, mandibular first molar was tender to percussion and the overlying buccal mucosa were sensitive to palpation; however, probing depth was not greater than 3mm. The distal half of the tooth had been replaced with large amalgam restoration. Radiographics showed an endodontically treated first molar with a large periapical lesion on the mesial root (Figure 1). Surprisingly, a large furcal perforation repair with amalgam was also evident. A diagnosis of chronic apical periodontitis was made. All adjacent teeth were sound. The possible treatment options were explained to the patient including i) tooth extraction with/without replacement, ii) endodontic retreatment, furcal perforation repair, crown lengthening, and post-core crown replacement, iii) periradicular surgery, and iv) intentional replantation. The patient rejected the first three treatment options due to financial limitations and was willing to maintain the tooth by any means. Therefore, IR was indicated with informed consent from the patient.

**Figure 2:**
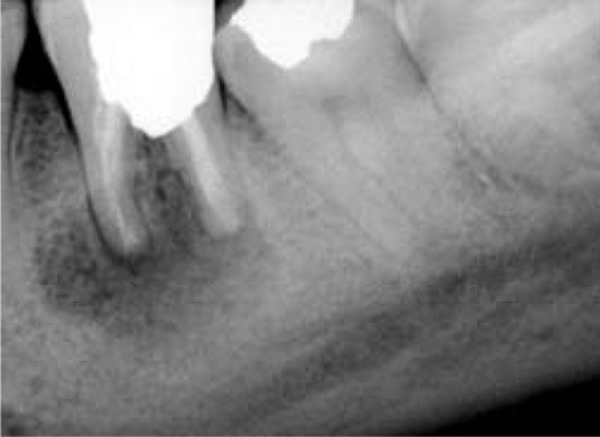
Immediate postoperative radiograph of the replanted tooth

Patient was prescribed 400 mg of Ibuprofen (Daroupakhsh, Tehran, Iran) a few minutes preoperatively to prevent postoperative pain. A 0.2% chlorhexidine rinse was carried out to control the oral microflora. After administering local anaesthesia (2% lidocaine with adrenaline 1:80000; Daroupakhsh, Tehran, Iran), the mandibular first molar was intentionally extracted without any damage to the buccal/lingual plates of the alveolar bone. After root-end resections, 3mm deep root-end cavities were prepared and the root-ends were filled using calcium enriched mixture (CEM) cement (BioniqueDent, Tehran, Iran). The tooth was then replanted into its alveolus; the accurate repositioning was confirmed radiographically (Figure 2). The whole procedure was carried out all in total of 6 minutes. The patient was given postoperative instructions for a soft diet and careful routine oral hygiene.

**Figure 3:**
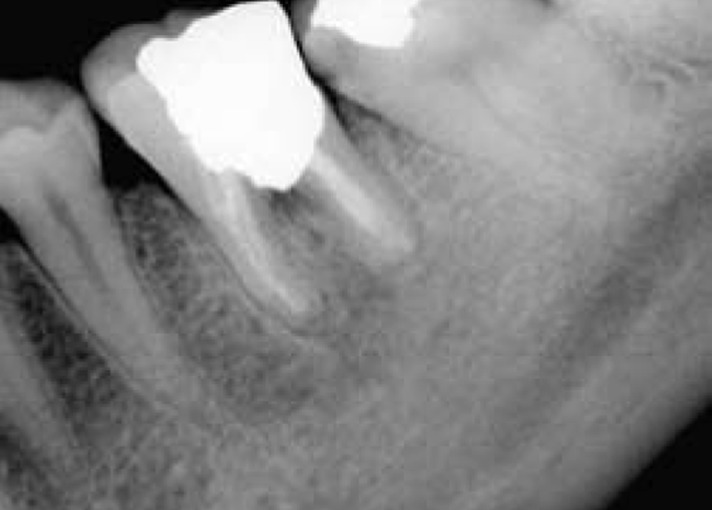
Two-year postoperative radiograph, PDL regeneration is evident.

The tooth was inspected 1, 7 and 14 days postoperatively via routine intraoral examinations. At 6, 12 and 24 months postoperative sessions there were no clinical sign/symptom of inflammation/infection, tenderness to percussion or palpation, pain or discomfort, mobility, and sinus tract formation; the periodontal examination showed normal sulcular depth and normal gingiva. Two-years radiographically follow-up revealed no pathological findings and showed normal periodontium; most importantly, the vertical and sagital dimensions of the alveolar bone remained unchanged (Figure 3)

## DISCUSSION

Intentional reimplantation may be conducted when routine RCT/endodontic surgery is impractical or impossible e.g. an obstruction of the canal [2][3]. This treatment has several advantages over endodontic surgery as it is less complicated, invasive, protracted, and expensive [14]. Case selection should be based on clinical and radiographic evaluations and must be performed warily to evaluate the risk of root fracture or damage to cementum/PDL during tooth extraction [12][13]. Before surgical intervention, the critical parameters e.g. root length/shape, amount of remaining bone/extent of osseous destruction, soft tissue attachment level, and patient’s oral hygiene were carefully evaluated. If case selection is carried out appropriately, the treatment’s ease and prognosis increase. In the present case, the patient’s chief complaints were failed endodontic treatment, chronic pain, and sensitivity to percussion and palpation. IR was chosen as the treatment option on the basis of the clinical/radiographic evaluation and also the patient’s refusal to have retreatment, periapical surgery, or tooth extraction. The two-year follow up confirmed the successful management of the case.

Endodontic literature has revealed a direct cause-and-effect relationship between treatment failure and presence of microorganisms and their by-products [15]. The success of this intentional reimplantation case was dependent on the maintenance of aseptic conditions during intervention, which was achieved through chlorhexidine mouthwash and disinfection of the operative field. Furthermore, the removal of all tissue debris and irritating substances from the root surface as well as achievement of a good apical seal by root-end resection, root-end preparation and root-end filling are necessary [16]. In the present case, a tight apical barrier was created with CEM cement which seals the pathways of communication between infected root canal system and the periradicular tissues.

Retention rate of IR teeth is reported to be ~50-95% [6][17][18][19]. Regeneration of the PDL is critical to the survival of the tooth, and ankylosis can result if the tooth is retained extraorally for a long period. Extraoral time (tooth outside of socket) should be kept to minimum to avoid dehydration and necrosis of ligament [18][20]. Careful avoidance of any form of trauma during extraction and reinsertion is also important for treatment success. Trauma to any of the tissues can become an additional cause of impaired healing. An atraumatic surgical technique preserves bone and periodontal support [13]. Atraumatic tooth extraction and the short extra-oral time (6 minutes) were important factors for success in the present case.

Periapical healing/periodontal health are more reliable factors for prognosis since slight external root resorption is usually not radiographically evident. Root resorption and replacement resorption (ankylosis) may be detectable within 3-4 weeks and 1-12 months, respectively [19][21]. A metallic sound when the tooth is percussed is, however, an accurate indication of tooth ankylosis [22]. No signs of ankylosis or inflammatory resorption were recorded during two-year follow up; PDL regeneration as well as absence of metallic percussive sounds in this case revealed favourable treatment outcomes.

## CONCLUSION

Intentional reimplantation is a treatment option which can be considered in the management of a hopeless tooth due to failed root canal (re)treatments. Annual clinical and radiographic follow ups should be carried out. More extensive studies are recommended.
